# Insertion variants missing in the human reference genome are widespread among human populations

**DOI:** 10.1186/s12915-020-00894-1

**Published:** 2020-11-13

**Authors:** Young-gun Lee, Jin-young Lee, Junhyong Kim, Young-Joon Kim

**Affiliations:** 1 Department of Integrated Omics for Biomedical Science, WCU Graduate School, Yonsei University, Seoul, Republic of Korea; 2 Department of Biochemistry, College of Life Science and Technology, Yonsei University, Seoul, Republic of Korea; 3 Department of Biology, University of Pennsylvania, Philadelphia, PA USA

**Keywords:** 1000 Genomes Project, Insertion, Structural variants

## Abstract

**Background:**

Structural variants comprise diverse genomic arrangements including deletions, insertions, inversions, and translocations, which can generally be detected in humans through sequence comparison to the reference genome. Among structural variants, insertions are the least frequently identified variants, mainly due to ascertainment bias in the reference genome, lack of previous sequence knowledge, and low complexity of typical insertion sequences. Though recent developments in long-read sequencing deliver promise in annotating individual non-reference insertions, population-level catalogues on non-reference insertion variants have not been identified and the possible functional roles of these hidden variants remain elusive.

**Results:**

To detect non-reference insertion variants, we developed a pipeline, InserTag, which generates non-reference contigs by local de novo assembly and then infers the full-sequence of insertion variants by tracing contigs from non-human primates and other human genome assemblies. Application of the pipeline to data from 2535 individuals of the 1000 Genomes Project helped identify 1696 non-reference insertion variants and re-classify the variants as retention of ancestral sequences or novel sequence insertions based on the ancestral state. Genotyping of the variants showed that individuals had, on average, 0.92-Mbp sequences missing from the reference genome, 92% of the variants were common (allele frequency > 5%) among human populations, and more than half of the variants were major alleles. Among human populations, African populations were the most divergent and had the most non-reference sequences, which was attributed to the greater prevalence of high-frequency insertion variants. The subsets of insertion variants were in high linkage disequilibrium with phenotype-associated SNPs and showed signals of recent continent-specific selection.

**Conclusions:**

Non-reference insertion variants represent an important type of genetic variation in the human population, and our developed pipeline, InserTag, provides the frameworks for the detection and genotyping of non-reference sequences missing from human populations.

**Supplementary information:**

**Supplementary information** accompanies this paper at 10.1186/s12915-020-00894-1.

## Background

Structural variants (SVs) are defined as genomic rearrangements with event sizes greater than 50 bp, including deletions, insertions, inversions, and translocations, relative to the reference genome [1]. As the human reference genome comprises few individuals of European ancestry and is represented as linear haploid sequences, comprehensive detection of SVs based on comparison of sequencing reads with the reference genome is limited [2]. Among SVs, long insertions (> 50 bp) relative to the reference genome are least well-identified owing to the ascertainment bias in the detection of variants that exist in the reference genome. Moreover, the complete sequences of the insertion variants are typically unknown in priori; therefore, reads originating from the insertion variants end up as unalignable. De novo assembly of unalignable reads may lead to better insertion mapping; however, the low complexity of typical sequences makes this challenging. In addition, insertion SVs represent a mixed set of novel insertions in the human genomes and retention of ancestral sequences in the reference genome [3]. Although the recent development of long-read sequencing has expanded the catalogue of long insertion variants [4], its applicability remains limited to a small number of individuals and not in population scale.

Non-reference insertion SVs have been classified as missing human genome sequences [5], novel sequence insertions [6], non-repeat non-reference (NRNR) sequences [7], or non-reference unique insertions [8], all of which represent insertion variants relative to the reference genome. The number of identified variants with breakpoint resolution ranges from 720 to 3791, which totals to 1.2–2.1 Mbp of additional segments [5, 7, 8]; 64–95% of non-reference insertion SVs are detected in non-human primate genomes, which suggests that these mostly represent deletions of ancestral sequences, including deletions from the reference genome [7, 8]. In a study that compared the human reference genome to the reference sequences of non-human primates, 571 non-reference insertion variants (1.55 Mbp) were segregated as biallelic among human populations [9]. Despite their potential importance, no population-level catalogue of non-reference insertion SVs has been compiled to date; therefore, the distribution of these variants among human populations and those that potentially affect adaptations and phenotypes remain unreported.

To systematically identify non-reference insertion SVs among human populations, we developed a pipeline, InserTag, which generates non-reference sequence contigs based on the local de novo assembly of unmapped reads and then infers the complete sequences from multiple reference genomes. By applying the pipeline to 2535 individual genomes from 26 populations from the 1000 Genomes Project (1KGP), we identified a set of 1696 high-confidence non-reference insertion SVs, of which 82.4% were traced to non-human primate genomes and the remaining were traced to other human genome assemblies. We observed that non-reference insertion SVs are common among human populations and more than half of the variants are major alleles, which indicates that the absence of the variants in the reference genome represents minor alleles. Among human populations, African populations have the greatest number of non-reference sequences owing to high retention of ancestral sequences compared to other continental groups. Our data also showed that the subsets of variants were in high linkage disequilibrium (LD) with phenotype-associated single nucleotide polymorphisms (SNPs), including those related to education attainment and age at menarche. Furthermore, certain variants showed signs of continent-specific selection in each continental group and were related to the genes with immunologic and metabolic functions, with the possible involvement of local adaptation. Addition of these non-reference insertion variants to the catalogue of human genetic variations could enhance our understanding of the variants that affect the phenotypes and adaptations among human populations.

## Results

### Discovery, tracing, and genotyping of non-reference insertion SVs

**Fig. 1:**
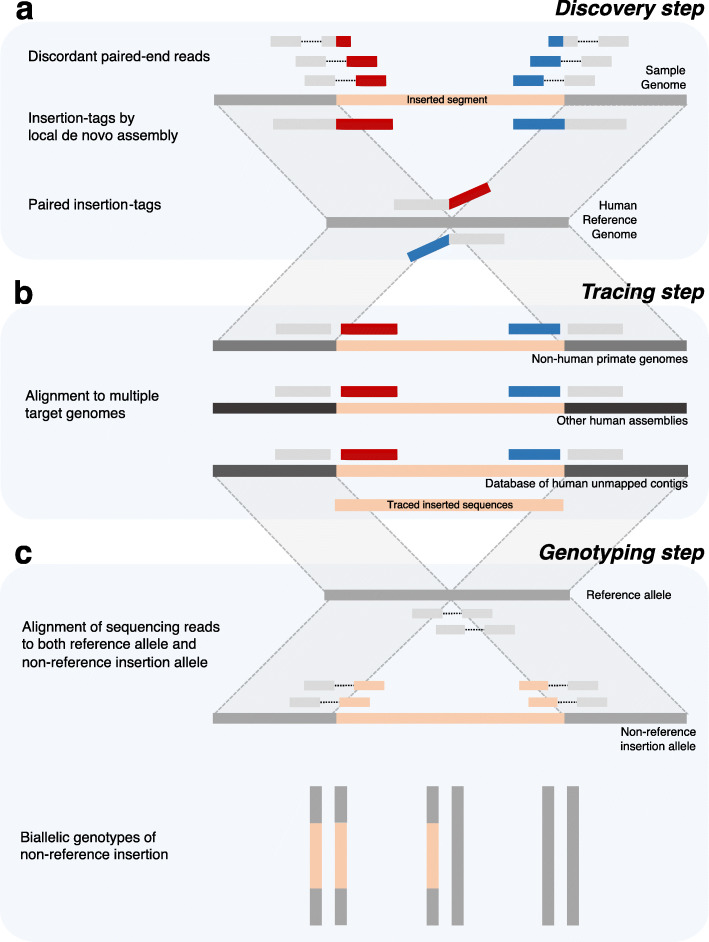
Overview of InserTag. **a** In the discovery step, discordant paired-end reads of the sample genome are clustered according to the location of anchored reads on the reference genome, and local de novo assembly is performed for each strand. The assembled contigs, called insertion-tags, consist of flanking reference sequences (gray bars), breakpoints, and partial inserted sequences (red and blue bars). If two insertion-tags are close and placed on opposite strands facing each other, then a putative insertion event is suggested to have occurred between the paired insertion-tags. **b** In the tracing step, each segment of paired insertion-tags is aligned to the target genomes, including non-human primate genomes, other human assemblies, and databases of human unmapped contigs, to trace the full insertion sequences. **c** Using the location of the insertion in the reference genome and the traced inserted sequences, both reference and non-reference insertion alleles are generated. The raw sequencing reads of the sample genome are aligned to these alleles, and the best-supported alleles are selected based on the alignment score. Using the read-depth ratio of supporting reads of each allele, the biallelic genotypes of non-reference insertion SVs are determined

To identify the sites of non-reference insertion SVs and the complete sequences of the inserted segments, we developed a pipeline, called InserTag, which is comprised of three steps: discovery, tracing, and genotyping. In the discovery step, a set of discordant paired-end reads (PERs), in which one end is anchored to the reference genome and the other end is unmapped or mapped discordantly, was selected. The PERs were clustered into a local set, and de novo assembly of these local reads was performed separately for each strand. The resultant contigs, which we have referred to as insertion-tags, consisted of local reference sequences, breakpoints, and partial insertion sequences. Two insertion-tags from opposite strands were paired if they were within two standard deviations of the mean insert length, which is suggestive of a putative insertion segment between the two insertion-tags (Fig. 1 (a)).

Paired insertion-tags are typically of adequate length to map uniquely to other genomes. In the tracing step, complete sequences of the inserted segments within the paired insertion-tags were inferred from non-human primate genomes or other human genome assemblies [3, 5] (Fig. 1 (b)). For tracing, the syntenic regions were identified using flanking reference sequences. Next, partial sequences of the inserted segments were aligned in conjunction with the flanking sequences. After the discovery and tracing steps, the insertion sites and full inserted sequences were catalogued. In the genotyping step, the genotypes of these catalogued insertion variants were computed from other sample genomes. Sequencing reads from sampled genomes were aligned to both the reference allele (without insertions) and the non-reference insertion allele. Based on the alignment score, the best-supported allele for each read was decided upon, and the genotype of the variant was calculated based on the read-depth ratio of the supporting reads (Fig. 1 (c), Additional file 1: Figure S1). As mutations around the breakpoint of each genotyped individual could alter the alignment score, in the genotyping step, we only used variants with lengths greater than the read length.

We validated the InserTag pipeline using three approaches. First, we simulated genomes with insertions of various lengths that were either unique or non-unique (i.e., comprising transposable elements and other repeat variations, see below) to the inserted genome. The sensitivity of detecting simulated insertion variants by the paired insertion-tags was 86.6% for unique insertions and 84.6% for non-unique insertions. The false discovery rate (FDR) was 0.5% for unique insertions and 1.2% for non-unique insertions, which was similar or superior to those of other similar local de novo assembly methods such as ANISE [10], MindTheGap [11], and PopIns [12]. In particular, our method was typically more than twice as sensitive to non-unique insertions (Additional file 1: Figure S2a and Additional file 2: Table S1). In terms of the accuracy of the identification of the breakpoints of simulated variants by the paired insertion-tags, InserTag had better performance than other methods, with approximately twice the number of precisely positioned breakpoints (Additional file 1: Figure S2b). Next, we applied InserTag to real sequencing data from eight individuals with non-reference insertion SVs confirmed by fosmid clone sequencing [5]. Among the 213 available clones with non-reference insertion SVs, 107 were discovered and traced by InserTag (sensitivity 50.2% = 107 clones out of 213). Manual confirmation by pairwise sequence alignment between the clones and the insertion variants reported by InserTag showed that all positive calls were matched with respect to the breakpoints and insertion sequences (FDR = 0%; Additional file 2: Table S2). Next, as the read-depth-based copy number states of the fosmid clones embedded with 31 non-reference insertion SVs from 26 individuals were available from the same study [5], we compared our genotype calls to the copy number states. Among 806 genotypes, 204 were undetermined owing to the low coverage of the sequencing data around the regions. Of the remaining 602 unequivocal genotype calls of 31 SVs, 557 calls matched with genotypes (92.5% sensitivity), and the FDR of the genotyping step was 7.5% (Additional file 1: Figure S3). The discrepancy between our genotype calls and the read-depth-based copy number states could be attributed to the genotyping of the heterozygote state, where InserTag favored homozygosity calls based on the alignment ratio around the breakpoints.

### Characteristics of non-reference insertion SVs in multiple populations

To catalogue the long insertion variants detected among the human populations studied, we applied InserTag to the genomic data of 2535 individuals from 26 populations included in the 1KGP, which included those of Africans (AFR), Americans (AMR), East Asians (ESA), Europeans (EUR), and South Asians (SAS) as aligned to hg19 in the 1KGP project [13]. In the discovery step of InserTag, a non-redundant discovered set of 7900 paired insertion-tags was generated. After the tracing step, we selected a final traced set comprising 1696 non-reference insertion SVs larger than 50 bp for further analyses (Additional file 1: Figure S4a). The compared genomes included non-human primate genomes (of chimpanzee, bonobo, gorilla, and orangutan), other human genome assemblies (HuRef and CHM1), and human unmapped contig databases (NRNR and GoNL; Additional file 2: Table S3). Both discovered and traced paired insertion-tags showed the ubiquitous distribution of variants over all chromosomes (Additional file 1: Figure S4b). Among 1696 insertion variants, 761 (44.9%) and 103 (6.0%) variants overlapped with the insertion SV and unresolved partial non-reference sequence set of the gnomAD database, respectively [14]. The remaining 832 (49.1%) insertion SVs were novel variants (Additional file 1: Figure S4c).

**Fig. 2:**
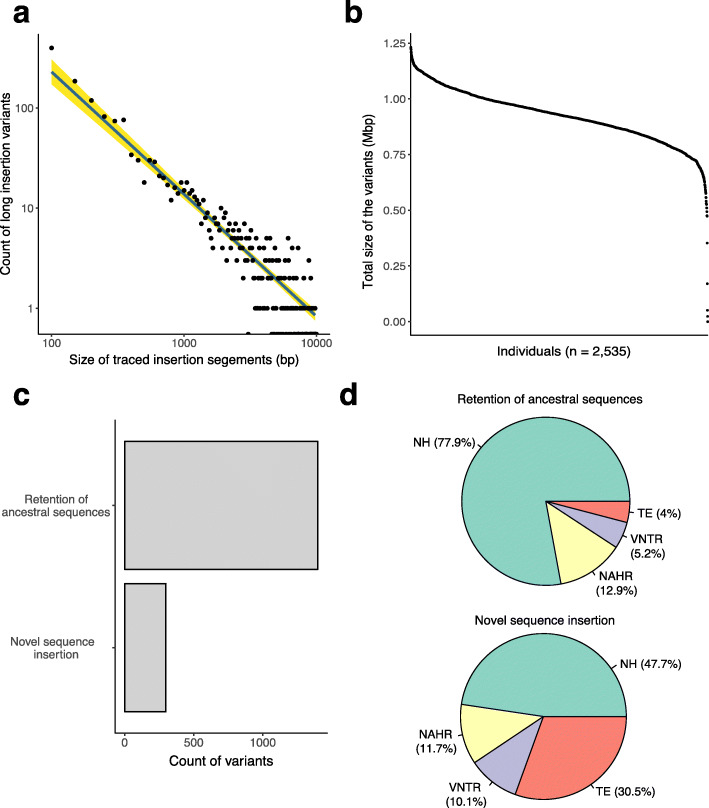
Catalogues of non-reference insertion SVs from the 1000 Genomes Project. **a** Size distribution of non-reference insertion SVs greater than 50 bp. The distribution indicates a significant negative relationship in the log-log linear regression model (*β* = − 0.93 s.d., *p* value < 2.0 × 10^−16^). The count and size of the variants are indicated on a log-10 scale, and variants larger than 10 kbp are omitted. **b** Total size of the genotyped insertion variants from each individual (*n* = 1148). Each dot represents an individual genome from the 1000 Genomes Project, arranged in decreasing size. **c** The number of variants in the retention of ancestral sequence and novel sequence insertions groups. **d** Proportion of inferred mutational mechanism between two groups of non-reference insertion SVs

The total size of the traced insertion segments was 1.86 Mbp, and the size distribution of the insertion variants was skewed to less than 100 bp and showed a negative linear correlation in a log-log regression model (*β* = − 0.93 s.d., *p* value < 2.0 × 10^−16^; Fig. 2a), which suggested the existence of a possible power-law relationship for the probability of an insertion of size *k*. In the genotyping step, we were able to genotype 1148 non-reference insertion SVs greater than 100 bp in length; we found that on average, individuals had 617 genotyped insertion variants spanning 0.92 Mbp (Fig. 2b).

The majority of the insertion variants were enriched in intergenic regions (61.3%) and introns (31.4%), while the remaining variants overlapped with non-coding RNAs (ncRNAs), untranslated regions (UTRs), and exons (Table 1). Gene ontology (GO) classification of the genes overlapping with or nearest to the insertion variants showed enrichment in functions related to the nervous system, including neurogenesis, neuron projection, and glutamatergic synapse (Table 2). To assess whether the non-reference insertion variants had unreported functional annotation, we used RNAseq data for the same 462 individuals from the 1KGP [15]. We searched for evidence of transcribed sequences by identifying the discordant PERs of RNAseq in which one end was anchored to the reference genome, and the mate-end, though previously unmapped, could be aligned to the insertion variants identified in our analysis. Among the 1696 non-reference insertion SVs, 195 variants (11.4%) were transcribed as modified transcripts, novel exons, or intergenic novel transcripts in the RNAseq datasets (Table 1).

**Table 1:** Annotations of the non-reference insertion SVs and novel functions revealed by RNAseq data

Annotation	Number	Novel function	Number
Exonic	3 (0.2%)	Modified transcript	3 (100%)
UTR	11 (0.7%)	Modified transcript	7 (63.6%)
ncRNA	109 (6.4%)	Modified transcript	22 (20.2%)
Intronic	533 (31.4%)	Novel exon	40 (7.5%)
Intergenic	1040 (61.3%)	Intergenic novel transcript	123 (11.8%)

*UTR* untranslated region, *ncRNA* non-coding RNA

**Table 2:** GO enrichment analysis of non-reference insertion SVs

GO term	Count	Fold enrichment	*p* value*
Cell adhesion (GO:0007155)	91	1.82	2.3 × 10^−3^
Neurogenesis (GO:0022008)	136	1.55	9.4 × 10^−3^
Glutamatergic synapse (GO:0098978)	49	2.46	9.0 × 10^−5^
Postsynaptic membrane (GO:0045211)	42	2.32	3.7 × 10^−3^
Cell junction (GO:0030054)	115	1.64	1.0 × 10^−3^
Neuron projection (GO:0043005)	113	1.57	8.8 × 10^−3^

*Bonferroni-corrected *p* values are denoted

We re-classified the non-reference insertion SVs by tracing to other genomes, as described above [3, 16]. If the traced sequences were found in non-human primate genomes, the discovered inserted sequences were determined to be retained ancestral sequences that were deleted from the genomes of the set of individuals comprising the human reference genome. If the traced sequences were detected only in other human genome assemblies and not in non-human primates, we inferred that the variants were generated by the insertion of novel segments after the split of humans from other primates. Among the 1696 non-reference insertion SVs, 1398 (82.4%) were traced to non-human primate genomes; we referred to this group as the retention of ancestral sequences group. We traced the remaining 298 (17.6%) variants only to other human genomes or to human unmapped contig databases; we referred to this group as the novel sequence insertions group (Fig. 2c).

To understand the mechanisms underlying the generation of non-reference insertion SVs, we used the breakpoint sequence features [3, 17] to classify the insertions into four categories. The variants were first annotated as variable number tandem repeats (VNTRs) and then defined as non-allelic homologous recombination (NAHR) based on the homologous features of the flanking sequences. The variants in which more than 80% of the sequences comprised retroelements, such as Alu, L1, or SVA, were defined as transposable element (TE) insertions. The remaining variants without recognizable features were classified as non-homologous (NH) events. Based on this classification, as expected within the novel sequence insertion group, TEs were present in a high proportion (Fig. 2d), and the most active subclasses of TEs, such as AluYb8, AluYa4, and L1HS [18–20], were observed frequently (Additional file 1: Figure S5a).

### Prevalence of non-reference insertion SVs among human populations

**Fig. 3:**
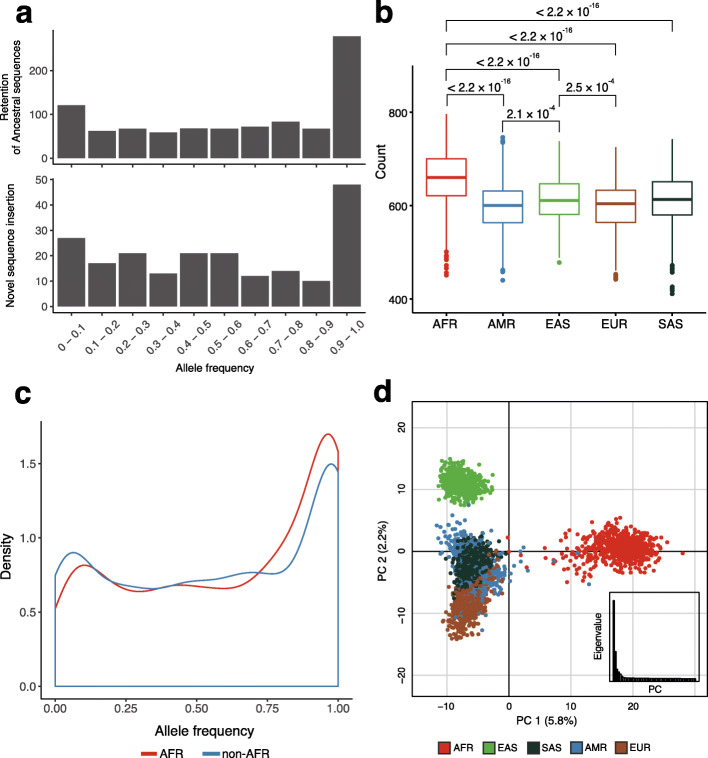
Prevalence of non-reference insertion SVs. **a** Allele frequency spectrum of the genotyped retention of the ancestral sequences (*n* = 944) and novel sequence insertions (*n* = 204) with respect to the ancestral status. **b** Number of variants from each individual, categorized according to the continental group. Significant *p* values of *t* test between the continental groups are indicated above the boxplot. **c** Allele frequency spectrum of the genotyped non-reference insertion SVs of African and non-African populations presented in a single group. **d** Principal component analysis using the biallelic genotypes of non-reference insertion SVs. Each dot represents an individual, and the colors indicate the continental groups. The first two principal components are plotted and the eigenvalues of each axis are plotted inset

Next, we analyzed the allele frequencies (AFs) in the retention of ancestral sequences group and the insertions of novel sequences group. We observed that 92.2% (*n* = 1059) of the variants were observed in more than 5% of human populations, which indicates that common insertion variants were missing from the reference genome (Fig. 3a). Moreover, 58.5% (*n* = 672) of the variants were greater than 50% in AF, which indicates that the absent status in the reference genome represents minor alleles [21]. When AFs were stratified based on the mechanisms, TEs in the novel sequence insertions group mostly had a low frequency, which indicates that active retroelements were abundant in TEs (Additional file 1: Figure S5b).

Of note, African populations had a significantly greater number of the insertion SVs (Fig. 3b; *p* value < 2.2 × 10^−16^). Among non-African populations, East Asian populations had more variants than European and American populations (Fig. 3b; *p* value = 2.5 × 10^−4^ and 2.1 × 10^−4^). The AF spectrum of the variants suggests that the high-frequency retention of ancestral sequences increased the genetic load of insertion variants in African populations (Fig. 3c). As African populations exhibited the most divergent AF spectrum compared to other population groups in the pairwise comparison of insertion variants (Additional file 1: Figure S6), we investigated the genetic differentiation based on a principal component analysis (PCA). The African and East Asian populations formed distinct clusters according to the first and second PCs, which accounted for 5.8% and 2.2% of the total variance, respectively (Fig. 3d). The European, American, and South Asian populations formed less distinct clusters in the PCA. A neighbor-joining tree based on population differentiation (*F*_ST_) showed clades consistent with the major geographic groups (Additional file 1: Figure S7). Therefore, we observed that the overall pattern of genetic differentiation in the genotyped insertion variants was similar to the pattern expected for the population level of differentiation at the SNP variation level [8].

### High LD between common non-reference insertion variants and phenotype-associated SNPs

**Fig. 4:**
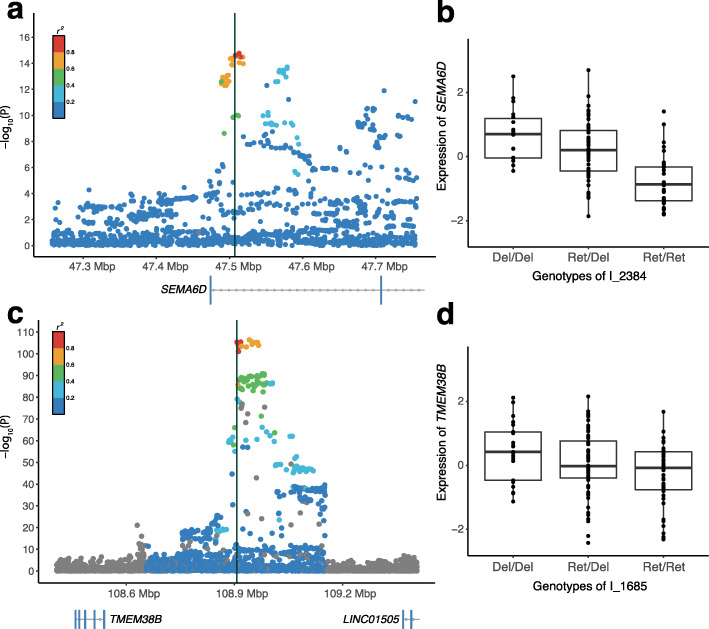
Regional association plots of phenotype-associated insertion variants. **a** Regional association plot of *15q21.1* for education attainment years in individuals with European ancestry. –log_10_(*p*) values of SNPs from previous studies are shown for the 250-kbp region on either side of the I_2384 variant. Each dot represents a SNP; the *r*^2^ values between the SNPs and I_2384 were computed based on data from the 1000 Genomes Project. **b** Expression of *SEMA6D* according to the genotypes of the I_2384 variant. **c** Regional association plot of *9q31.2* for the onset of menarche in European populations. **d** Expression of *TMEM38B* according to the genotypes of the I_1685 variant

As the current reference genome limits the representation of common long insertion variants that are prevalent among human populations, new phenotype associations could be detected by the inclusion of non-reference insertion variants in genome-wide association studies (GWAS). Using a window size of 250 kbp, we observed a high LD (*r*^2^ > 0.6) between 72 non-reference insertion SVs and 87 phenotype-associated SNPs reported in GWAS Catalog [22] (Additional file 2: Table S4). Compared to the variants reported in the 1KGP, GWAS-associated non-reference insertion SVs (6.4%) were similar to biallelic SNPs (5.2%), indels (6.1%), or SVs except duplications (0.3%, *p* < 0.001) (Additional file 2: Table S5). Among these, the I_2384 variant located in the intronic region of *SEMA6D* had a high LD with the SNPs associated with the education attainment phenotype [23] (Fig. 4a). *SEMA6D* is expressed during brain development and is related to neural circuits [24] and axonal guidance [25]. The retention of ancestral sequences was related to a reduction in education attainment years and associated with the downregulation of the *SEMA6D* in brain tissue (see the “Methods” section; *β* = − 0.70, SE = 0.11, *p* value = 6.2 × 10^−9^; Fig. 4b).

A second example of interest is the I_1685 variant 367 kbp downstream of *TMEM38B*, which had a high LD with SNPs associated with the age at menarche phenotype in European populations [26] (Fig. 4c). As the closest gene, *TMEM38B*, has multiple expression quantitative trait loci (eQTLs) in brain tissue, as observed in the Genotype-Tissue Expression (GTEx) project [27], we postulated that the I_1685 variant may delay menarche by affecting the expression of *TMEM38B* in a brain-specific manner. The retention of ancestral sequences was associated with the downregulation of the expression of the corresponding gene in brain tissue (see the “Methods” section; *β* = − 0.14, SE = 0.05, *p* value = 3.9 × 10^−3^; Fig. 4d), whereas it was not associated with the same in lymphoblastoid cells.

As shown in the examples, 27 neuropsychiatric phenotypes (31%) exhibited a high LD with the non-reference insertion variants. To further evaluate if the insertion variants were related to variations in the transcriptome of brain tissues, we first selected 543 pairs of non-reference insertion SVs and eQTLs from the GTEx project with high LD (*r*^2^ > 0.8) and clustered the pairs based on the *m* value [28], which represents the posterior probability of eQTLs exerting effects in multiple tissues. Among various tissues, 25.7% of the insertion variant-eQTL-gene pairs were effective (*m* value > 0.9) in brain tissues [29] and 3.4% were effective exclusively in 13 brain tissues (Additional file 1: Figure S8). These results suggested that a subset of non-reference insertion SVs could exert putative functional effects on neuropsychiatric phenotypes by affecting the transcriptome of brain tissues.

### Population stratification of the retention of ancestral sequences

**Fig. 5:**
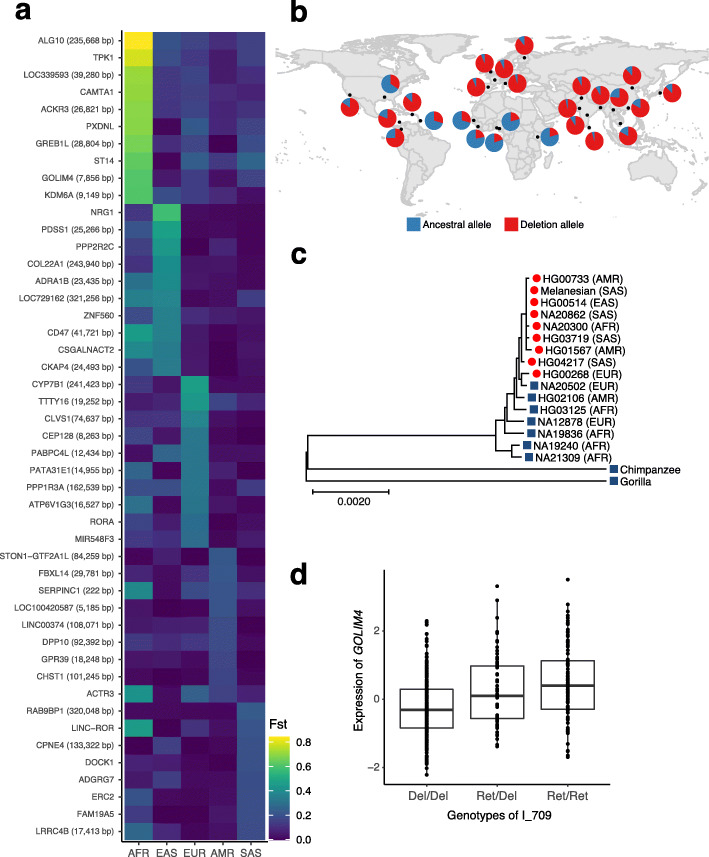
Population-stratified retention of ancestral sequences. **a** Top 10 *F*_ST_ signals in each continental group. Each row represents the non-reference insertion variants and the nearest genes within the 250-kbp range are listed with distances mentioned in parentheses. Each cell is colored according to the *F*_ST_ value. **b** Global distribution of I_709. **c** An unrooted maximum-likelihood tree based on the alignment of 30-kbp flanking sequences of I_709 from 16 human assemblies. Assemblies with ancestral sequences are indicated by blue boxes and those with deletion alleles by red circles. **d** Expression of *GOLIM4* according to the genotypes of the I_709 variant

To identify the variants that underwent recent continent-specific directional selection, we computed pairwise population differentiation (*F*_ST_ [30]) for each population as a part of the total diversity [31]. Genes that overlapped or were closest to the variants with top 10 *F*st in each population (Fig. 5a) were related to the functions which have undergone recent adaptations in humans [32, 33] (Fig. 5a and Table 3).

**Table 3:** Genes related to the population-stratified retention of ancestral sequences

Functional category	Genes overlapping/closest to the variants
Metabolic process	*ALG10*, *ATP6V1G3*, *CHST1*, *CSGALNACT2*, *PPP13RA*, *TPK1*
Immunologic function	*ACTR3*, *CD47*, *CKAP4*, *CYP7B1*, *DOCK1*, *RORA*, *SERPINC1*

One of the population-stratified variants of interest was a 1170-bp ancestral sequence prevalent in African populations (I_709; AF = 0.93) and mostly deleted in non-African populations, which was located approximately 8 kbp upstream of *GOLIM4* (Fig. 5b). Alignment of the 30-kbp sized flanking sequences without the insertion sequences from 16 assembled human genomes verified that alleles with the insertion variant are ancestral to the alleles without the variant (Fig. 5c). To determine the functional impact of the variants, we searched for evidences of the regulation of the downstream gene *GOLIM4* in lymphoblastoid cells. The deletion of ancestral sequence variant was significantly associated with the downregulation of *GOLIM4* expression (see the “Methods” section; *β* = − 0.21, SE = 0.03, *p* value = 1.8 × 10^−10^; Fig. 5d). Furthermore, this association was consistent among haplotypes with insertion variants (Additional file 1: Figure S9). As the GOLIM4 provides the infectious protein Shiga toxin with a means of escaping the degradation system of the late endosome, downregulation of the *GOLIM4* may result in the accumulation of Shiga toxin in the early endosome, which would consequently help inhibit infection [34, 35]. Collectively, the widespread distribution of ancestral sequence retention in non-African populations may have resulted from adaptations to environmental changes by increasing resistance to infectious toxins.

## Discussion

We developed InserTag, which is a pipeline for detecting non-reference sequences using short-read sequencing data. Compared to previous methods, InserTag implemented strand-specific local de novo assembly, to generate non-reference contigs without pre-defined insertion contents. The contigs were then traced to multiple genomes, including those of humans and non-human primates, to detect non-reference sequences. Owing to the possibility of errors and representation of minor alleles, a single reference genome is considered inadequate and biased for the detection of SVs, especially non-reference sequences. Recent studies have suggested the existence of multiple alternative sequences among human populations [8], and approximately 15% of the variants are considered to be shared across all samples or presented in more than half of the samples [4], which indicates the importance of generating pan-genomes [36, 37] or population-specific consensus genomes [38]. In this study, we used multiple reference genomes to identify the complete insertion sequences that are not represented in the reference genome, which otherwise yielded unresolved sequences or underestimated short contigs when compared against a single reference.

By application of InserTag to the 1KGP dataset, we could generate a comprehensive catalogue of insertion SVs relative to the reference genome. Compared to those in the gnomAD database, approximately half of the variants were novel and 6% were completely resolved by InserTag. Although the gnomAD database represents 433,371 SVs from 14,891 individual genomes, the discovery pipeline of gnomAD is based on methods such as Manta [39], DELLY [40], MELT [41], and cn. MOPS [42], which are not designed to detect non-reference sequences. Therefore, InserTag could be applied to a greater number of databases to further catalogue non-reference insertion variants among human populations.

The majority of newly discovered variants from the 1KGP were common among human populations, and more than half of the variants were major alleles, which indicates that the current reference genome represents minor alleles. Although catalogues of long insertion variants from African populations [36] and Icelandic populations [7] have been reported, we identified these non-reference variants in one of the largest and most frequently studied population-scale genome datasets. The population-level analysis of insertion variants in this study confirmed earlier findings. First, we revealed that these variants are widespread among human populations and that African populations had the largest number of non-reference sequences. This is expected, as rare and singleton insertion variants are abundant in African populations [4, 36]. Second, the retention of ancestral sequences of high frequency was highly prevalent in African populations. This result is consistent with that of a previous study that suggested that African populations retain more ancestral sequences, which can lead to the misinterpretation of the unbalanced SV loads between African and non-African populations [9]. Third, the variants exert putative functional effects by introducing structural changes to genes or by modifying regulatory elements [43, 44].

We re-classified the variants based on the ancestral states and found that 82.4% of the variants were categorized under the retention of ancestral sequences group. This group represents the deletion variants compared to the ancestral states, with the deletion event occurring in the reference genome. The remaining 17.6% of the variants were categorized under the novel sequence insertions group as the variants were detected in humans, whereas they were not detected in non-human primates. TEs were abundant in this group, and most of them were classified as active retroelements, such as AluY and L1HS. Consistent with this, the AFs of the TEs in this group are mostly in low. Conversely, the AFs of other mechanisms, such as NAHR, VNTR, and NH, are mostly fixed, which indicates that the errors or rare non-insertion alleles are present in the reference genome.

As shown in our dataset, 72 common non-reference insertion variants had a high LD with phenotype-associated SNPs from previous GWAS. Compared to variants reported by the 1KGP, the ratio of GWAS-association of non-reference insertions was similar to SNPs, indels, and SVs, whereas it was not similar for duplications. The depletion of linkage between duplications and GWAS-associated SNPs is attributed to the gap between the duplicated sites and the genomic locations where the duplications are inserted [45], which indicates the importance of the detection of insertion sites in the functional analysis of SVs [43]. By adding the insertion variants into the current catalogue of SVs, we could enhance the potential for detection of novel phenotypic associations; additionally, certain variants were observed to perform putative regulatory functions by affecting the expression of the corresponding genes. Of note, 31% of the associations were related to neuropsychiatric phenotypes, and the total non-reference insertion variants were enriched for the functional annotations of the nervous system. As the insertion or deletion SVs are associated with certain Mendelian neurologic and psychological disorders [46], the non-reference insertion variants could exert similar effects.

A subset of ancestral sequences showed continent-specific stratification, which suggests the occurrence of recent positive selection events. These insertion variants overlapped with genes related to immunologic and metabolic functions, which are known to be adaptive among human populations and are affected by CNVs [44]. One of the strongest signals of population stratification was associated with immunologic function. A 1-kbp sized intergenic insertion variant regulates *GOLIM4* expression. As low expression levels of this gene are associated with the susceptibility to the infectious agents, we hypothesized that the deletion of the insertion variant could lead to functional advantages against infectious diseases.

Although we conducted a genome-wide assessment of non-reference insertion SVs from multiple populations, most of our paired insertion-tags remained unanalyzed. The discovery set, in which the closest insertion-tags within a certain range of distance are paired, reduced to one-tenth after the variants from non-human primate genomes or other human genome assemblies were traced (Additional file 1: Figure S4b). As we selected this strategy to identify the full inserted sequences without determining presumed size or content of the insertion variants, singleton variants were inherently deprived in the resulting traced set. This ascertainment bias could be the reason for the enrichment of non-reference insertion variants reported in this paper in the common alleles and their distinction from the long-read sequencing data in which individuals have abundant singleton variants [4]. However, singleton or rare variants identified in a small number of individuals can be identified as common alleles based on population-scale genome data, such as the 1000 Genomes Project.

## Conclusion

The non-reference insertion variants represent an important type of genetic variation in the human population, and their extent and functional impact among human populations remain to be explored. Although catalogues of these missing sequences are expected to be increased by the application of the long-read sequencing, our approach could be applied to the abundant short-read sequencing data with population scale, not only for human data but also for other organisms.

## Methods

### Process followed by InserTag

#### Discovery step

BAM-formatted alignment files of paired-end sequencing data were used as the input for InserTag. PERs in which only one end is anchored to the reference genome and the other end is unalignable or aligned in discordant locations were selected. Next, these PERs were clustered first by the strand of anchored reads and then by the location between anchored reads at which the distance between the reads was within two standard deviations from the mean insert length of the PER library. In each cluster of PERs, contigs were generated using both anchored and overhang reads simultaneously to span the breakpoints within the contigs. The *Velvet* algorithm [47], based on the De Brujin graph, was used for local de novo assembly. To reduce the number of falsely assembled contigs, the location and strand of each read within the contigs were tracked if all member reads suggested identical events but an artificial assembly. A contig was divided into the insertion and reference parts, and the breakpoint was defined as the phase-changing point of the two parts within the contig. For this purpose, the mapping portion of the reads was masked using the CIGAR string, which is the code for the alignment status. Next, the contig was re-aligned to the local reference genome using *Blat* [48]. After the breakpoint refinement step, the contigs were denoted as “insertion-tags” and divided into flanking reference sequences and inserted segment sequences. Because the assembly step was performed in a strand-specific manner, the insertion-tags were anchored to the reference genome by the strand of anchored reads. If two insertion-tags were derived from an identical insertion variant, the upstream and downstream insertion-tags could be paired relative to the specific breakpoint and were denoted as “paired insertion-tags.” For pairing, each left-flanking insertion-tag was scanned against the right-flanking insertion-tags within two standard deviations from the mean insert length of the PER library.

#### Tracing step

Although the paired insertion-tags could suggest putative insertion variants, the rate of false positives could be high because the insertion-tags were paired even though the two insertion-tags were not in the same allele. Moreover, the paired insertion-tags represented the two boundary segments of insertion events; the complete insertion segments remained unknown. To overcome these limitations, the tracing concept was used to further define the confident set of paired insertions and to fully infer the insertion sequences. Each segment (e.g., flanking reference and insertion segment) of paired insertion-tags was aligned separately to the other target genomes using *BWA-MEM* [49]. For proper tracing, alignment was performed in a continuous manner. First, the flanking reference sequences were aligned to locate the syntenic regions, following which the insertion segments were aligned to those regions. When insertion events were traced to more than one target genome, multiple sequence alignments were generated to determine whether all the tracings matched. After the tracing step, the putative insertion variants with insertion sites and the complete inserted sequences were catalogued.

#### Genotyping step

To genotype a putative insertion variant from other sample genomes, two sequences of the variant were generated: (i) an insertion variant allele, which was generated by concatenating reference sequences flanking the breakpoint and inserted segments, and (ii) local reference sequences spanning the breakpoint. The local reference sequences extended for 50 bp from the insertion breakpoints. Next, the raw sequencing reads of sample genomes were aligned to the two target sequences. Reads that unequivocally selected one target sequence were selected and counted. The read-depth ratio of the reads that supported the insertion variant allele and reference allele was then used to determine the genotype of the variant.

### Validation using a simulated dataset

Simulated insertion segments were generated by randomly selecting segments of sizes ranging from 100 bp to 100 kbp from chromosome 16 of the reference genome (hg19). The segments were randomly inserted into chromosome 17 of hg19. PERs were generated with various sequencing coverages and read lengths using *wgsim* [50]. To test the performance of InserTag in cases with unique and non-unique insertions, the reads were first mapped solely to chromosome 17 as a reference, and then to both chromosomes 16 and 17 as the new references, using *BWA*. The performance of InserTag was compared to those of ANISE [10], MindTheGap [11], and PopIns [12] using the default parameters. The reported breakpoints and insertion sequences were extracted from the output files of each method. To ensure precision of the tools used for breakpoint detection, the gaps between the reported and simulated breakpoints were considered. If the gaps spanned for less than 100 bp, the predicted insertion sequences were aligned to the simulated insertion segments using *Blat*. Positive calls were made when at least 50% of the predicted insertion sequences were aligned to the simulated sequences.

### Validation using fosmid clones with non-reference insertion SVs

The breakpoints and full sequences of insertions from the genomic data of nine individuals with non-reference insertion SVs confirmed by fosmid clones were available from a previous study [5]. Among these, sequencing data for eight individuals were available from the 1KGP. InserTag was applied to the sequencing data, and the results were compared with the sequences of the fosmid clones by pairwise sequence alignment. The read-depth-based copy number states of 31 non-redundant sequenced clones from 26 individuals were also available from the same study. The genotype calls of InserTag for the same 26 individuals from the 1KGP were compared with the reported copy number states.

### Application of InserTag to 1KGP data

1KGP phase 3 sequencing data aligned with the reference genome (hg19) in BAM format were downloaded from [51]. The files were first sorted by the name of the reads, and each PER was marked as an anchored or overhang read. The PERs with low mapping qualities of anchored reads were excluded. For each individual genome, the mean length of sequencing reads and library inserts was calculated. Three steps of InserTag were performed using PERs from 2535 individuals from 26 populations. For the tracing step, multiple genomes, including those of chimpanzee (panTro5), bonobo (panPan2), gorilla (gorGor5), orangutan (ponAbe2), HuRef [52] (GCA_000002125.2), and CHM1 [53] (GCA_001297185.2), were used. For databases of unmapped human contigs, including NRNR [7] and GoNL [54], the data were retrieved from two studies. For the genotyping step, non-reference insertion SVs larger than 100 bp were selected. The abbreviations used for each population and continental group were according to those recommended in the International Genome Sample Resource: AFR, Africans; EAS, East Asians; SAS, South Asians; AMR, Americans; EUR, Europeans; ACB, African Caribbeans in Barbados; ASW, Americans of African ancestry; BEB, Bengali from Bangladesh; CDX, Chinese Dai in Xishuangbanna of China; CEU, Utah residents with Northern and Western European ancestry; CHB, Han Chinese in Beijing; CHS, Southern Han Chinese; CLM, Colombians from Medellin; ESN, Esan in Nigeria; FIN, Finnish in Finland; GBR, British in England and Scotland; GIH, Gujarati Indian from Houston; GWD, Gambian in western divisions in the Gambia; IBS, Iberian population in Spain; ITU, Indian Telugu from the UK; JPT, Japanese in Tokyo; KHV, Kinh in Vietnam; LWK, Luhya in Kenya; MSL, Mende in Sierra Leone; MXL, Mexican ancestry from Los Angeles; PEL, Peruvians from Lima; PJL, Punjabi from Pakistan; PUR, Puerto Ricans; STU, Sri Lankan Tamil from the UK; TSI, Toscani in Italia; and YRI, Yoruba in Ibadan of Nigeria.

### Functional annotation of non-reference insertion SVs

The non-reference insertion SVs were annotated based on the breakpoint location of the refGene of hg19. For the GO enrichment analysis, genes that were overlapping, or closest to, the variants were used as inputs for PANTHER [55].

### RNAseq data analysis

Raw RNAseq data for 462 individuals from the 1KGP were downloaded from EBI ArrayExpress under the accession ID *E-GEUV-1* [15]. The PERs in which only one end was mapped were selected. The unmapped end-reads were re-aligned to those non-reference insertion SVs around the mapped end-reads. The updated annotations were defined based on the annotations of the mapped end-reads and non-reference insertion SVs. For novel exons, the mapped end-reads were located in the known exons and the insertion sequences were located in introns. For unannotated transcripts, the mapped end-reads and insertion sequences were located in the intergenic regions or introns. For modified transcripts, the mapped end-reads and insertion sequences were located in the UTRs or exons.

### Inferring the mutational mechanism using breakpoint sequence features

Mutational processes, including NAHR, VNTR, TE, and NH events, were inferred using both flanking and insertion sequences, as suggested by BreakSeq [3, 17]. The flanking reference sequences located 100 bp upstream and downstream of the location of the non-reference insertion SVs were extracted.

### Population genetic analysis

In each genotyped variant, pairwise *F*_ST_ value between each continental group and all other continental groups based on the methods described by Nei [30]. For functional annotation, the top 10 *F*_ST_ hits from each continental group were selected. PCA was performed using the biallelic genotypes of the variants. These analyses were performed using the R package *heirfstat*.

### GWAS-associated non-reference insertion SVs

To calculate LD between non-reference insertion SVs and SNPs, 1KGP phase 3 genotypes data were downloaded from [51]. The LD between non-reference insertion SVs and SNPs within 250 kbp was computed using PLINK [56]. The list of phenotype-associated SNPs reported by NHGRI-EBI [22] (v1.0.2 2020-03-08) was downloaded from [57]. For comparison, the LD between SNPs, indels, or SVs from the final report of the 1KGP and phenotype-associated SNPs were calculated. Summary statistics from each GWAS were retrieved from the Social Science Genetic Association Consortium and The ReproGen Consortium.

### Phylogenetic analysis

A haplotype structure was constructed using 18 SNPs within the LD structure of the region encompassing the insertion variant. Haplotype network analysis was performed using PopART [58] by the median-joining method. From the reference genome (hg19), sequences of the 30-kbp flanking region of the insertion variant were retrieved and used as a query to identify the corresponding regions from 16 human assemblies available in the NCBI Assembly database. Evolutionary history was inferred using the maximum-likelihood method and the Kimura 2-parameter model with the complete-deletion option from MEGA X [59].

### Microarray data analysis

Transcription profiling data from the microarray analyses of lymphoblastoid cells from HapMap 3 were available from EBI ArrayExpress under the accession identifier *E-MTAB-198* [60] and *E-MTAB-264* [61], and 527 individuals were also target samples of 1KGP. The population structures for each individual were calculated using SNPs and were used as covariates in linear regression. The expression and genotyping data of various brain tissues are available from the UK Brain Expression Consortium. The genotype data was phased using SHAPEIT [62] and imputed for the insertion variants using IMPUTE2 [63]. Linear regression of the genotypes of non-reference insertion SVs and rank-normalized expression levels of the target genes were computed using R.

## Supplementary information

Additional file 1: **Figure S1.** Genotyping step of InserTag. **Figure S2.** Performance of InserTag compared to other methods. **Figure S3.** Validation of the genotyping step in InserTag. **Figure S4.** Number of variants in each step of InserTag. **Figure S5.** Distribution of TE subclasses in novel sequence insertion group. **Figure S6.** Pairwise correlation matrix of allele frequencies of non-reference insertion variants among human populations. **Figure S7.** Phylogenetic tree based on non-reference insertion variants. **Figure S8.** Tissues affected by the eQTLs linked to non-reference insertion SVs. **Figure S9.** Haplotype association analysis of I_709 and *GOLIM4*. (DOCX 1.55 mb)

Additional file 2: ** Table S1.** Sensitivity and false discovery rate of InserTag, ANISE, MindTheGap and PopIns on simulated data. **Table S2.** Validation of InserTag by fosmid-clone confirmed insertion variants. **Table S3.** Number of paired insertion-tags traced to target genomes. **Table S4.** Phenotype-associated SNPs with high linkage disequilibrium with non-reference insertion variants. **Table S5.** Ratio of high linkage disequilibrium between phenotype-associated SNPs and non-reference insertion variants. (XLSX 38.7 kb)

## Data Availability

All data generated or analyzed during this study are included in this published article, its supplementary information files, and publicly available repositories. Insertion calls with genotypes of the 1000 Genomes Project samples are available under dbVar accession *nstd194*. Source code for InserTag can be downloaded from [64].
