# Supplementary material for: Insertion variants missing in the human reference genome are widespread among human populations
Source: BMC Biol. 2020 Nov 13;18:167. doi: 10.1186/s12915-020-00894-1 (PMC7666470; doi:10.1186/s12915-020-00894-1)
Supplement: Supplementary file 1 — Figure S1. Genotyping step of InserTag. Figure S2. Performance of InserTag compared to other methods. Figure S3. Validation of the genotyping step in InserTag. Figure S4. Number of variants in each step of InserTag. Figure S5. Distribution of TE subclasses in novel sequence insertion group. Figure S6. Pairwise correlation matrix of allele frequencies of non-reference insertion variants among human populations. Figure S7. Phylogenetic tree based on non-reference insertion variants. Figure S8. Tissues affected by the eQTLs linked to non-reference insertion SVs. Figure S9. Haplotype association analysis of I_709 and GOLIM4. (DOCX 1.55 mb) [file 12915_2020_894_MOESM1_ESM.docx]

**Figure S1. Genotyping step of InserTag.**

**
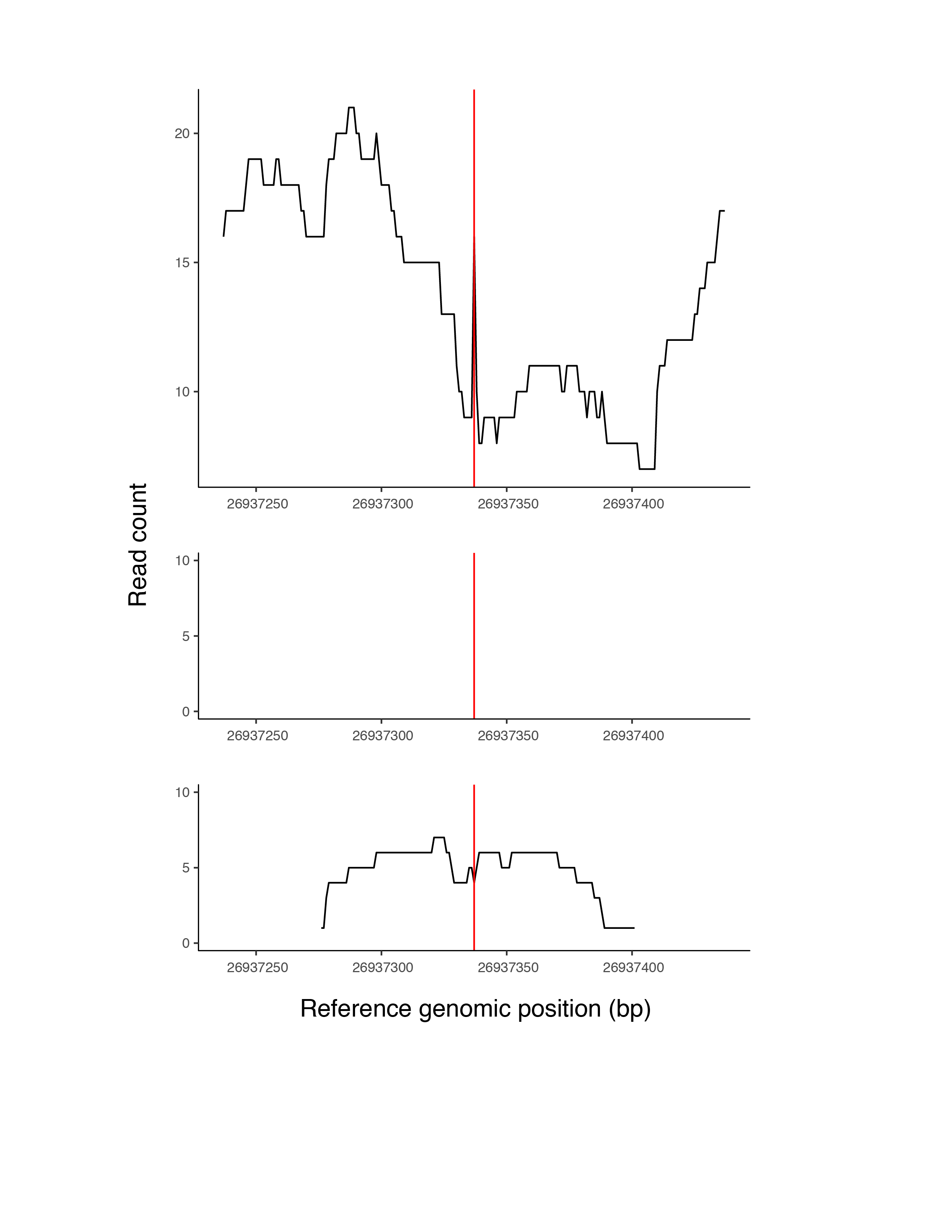
**

Top: Read depth of all paired-end reads of NA18939 around the variant of I_371 (red solid line). Middle: Read depth of discordant paired-end reads supporting the reference allele without the insertion variant. Bottom: Read depth of discordant paired-end reads supporting the non-reference allele which I_371 (length = 2,416 bp) is inserted within the reference genome.

**Figure S2. Performance of InserTag compared to other methods.**


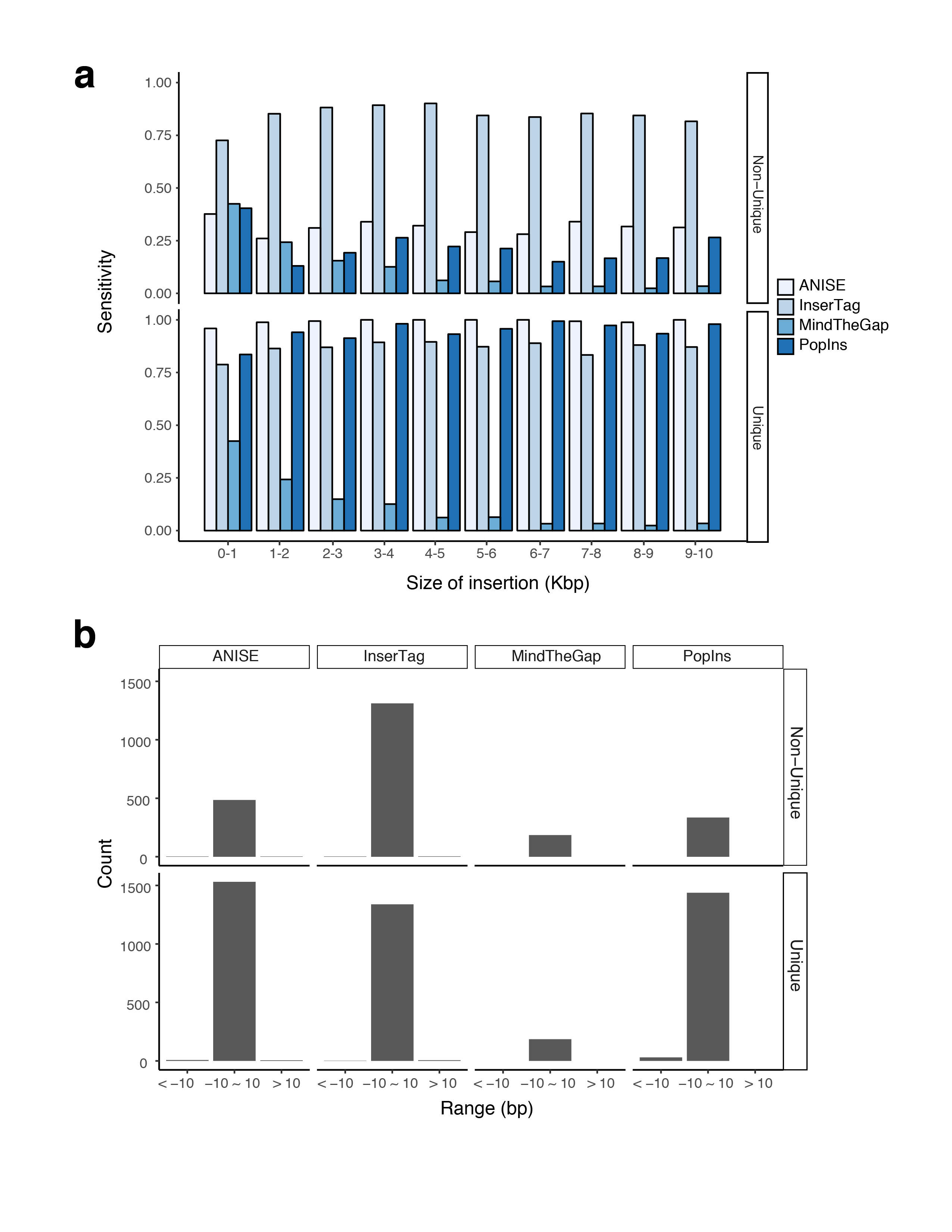


(a) Sensitivity of InserTag, ANISE, MindTheGap, and PopIns in the detection of simulated unique and non-unique insertions. (b) Gaps between the simulated breakpoints and the called breakpoints in each method.

**Figure S3. Validation of the genotyping step in InserTag.**


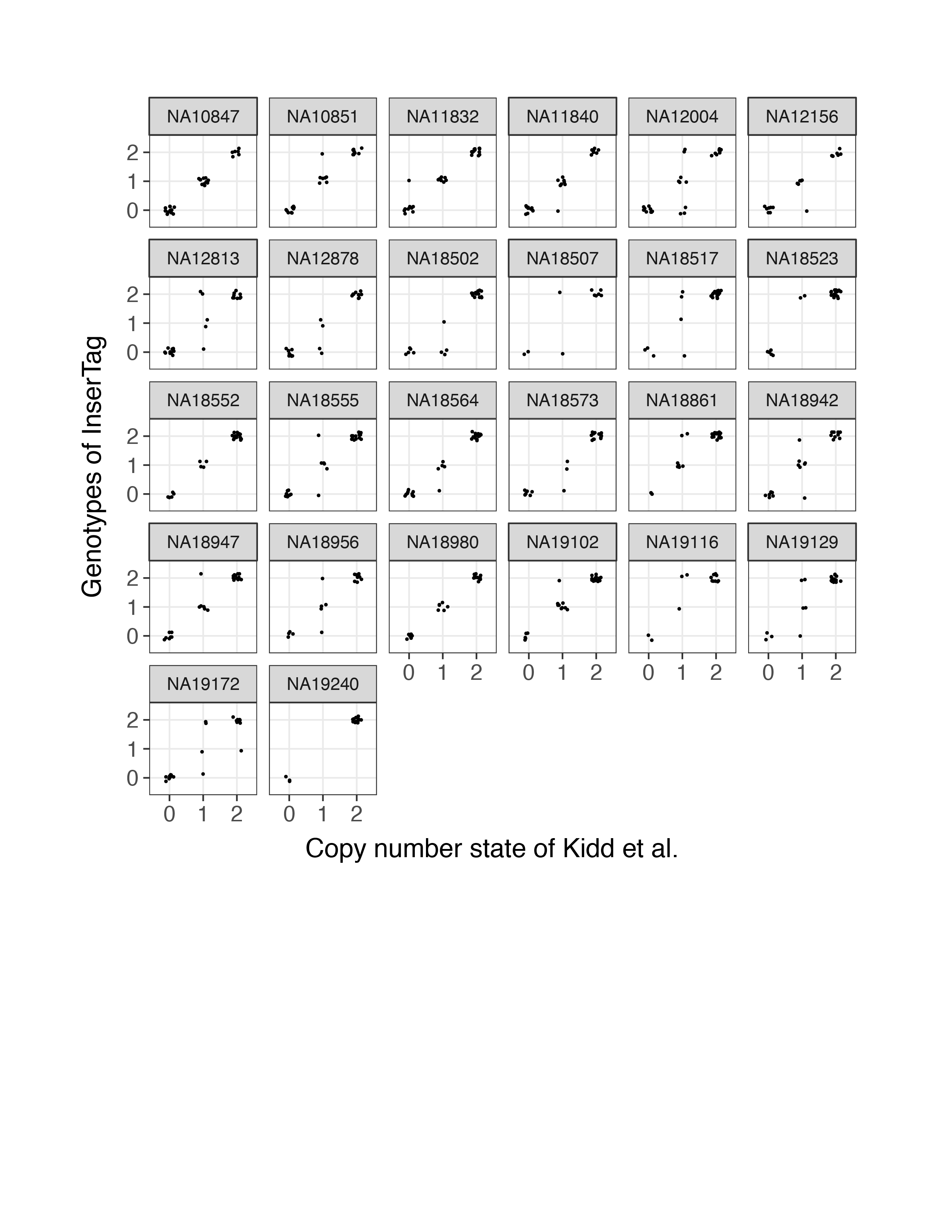


Agreement between the genotypes determined by InserTag and the read-depth based copy number states determined by Kidd et al. Each box represents 26 individuals from the 1000 Genomes Project and each dot represents the genotypes of 31 non-reference insertion variants after exclusion of the unequivocal genotype calls.

**Figure S4. Number of variants in each step of InserTag.**


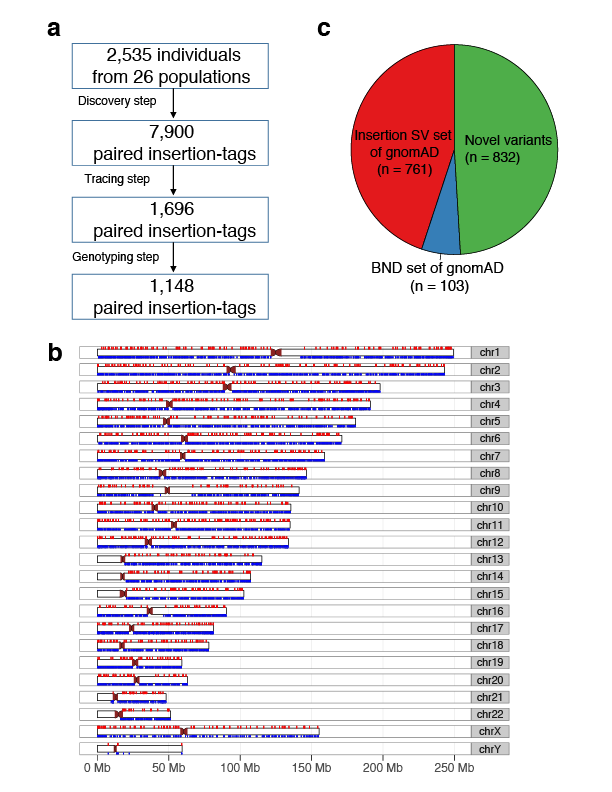


(a) Workflow and number of total variants in the three steps of InserTag. (b) The discovery set of InserTag (n = 7,900) is indicated by the blue bar under the ideogram. The traced set (n = 1,696) is indicated by the red bar above the ideogram. (c) Overlap between the insertion SV and the unresolved breakend sequence (BND) sets of the gnomAD dataset and the traced set of InserTag.

**Figure S5. Distribution of TE subclasses in novel sequence insertion group.**

**
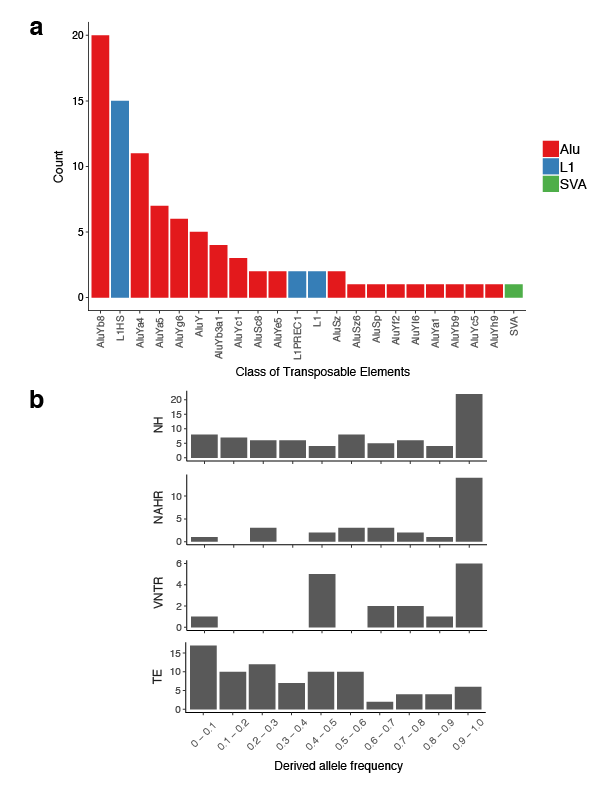
**

(a) Count of each TE class in the novel sequence insertion groups. (b) Allele frequency spectrum of the novel sequence insertion groups stratified according to the mechanisms of insertion. NH, Non-homologous event; NAHR, Non-allelic homologous recombination; VNTR, Variant number of tandem repeats; TE, Transposable element.

**Figure S6. Pairwise correlation matrix of allele frequencies of non-reference insertion variants among human populations.**

**
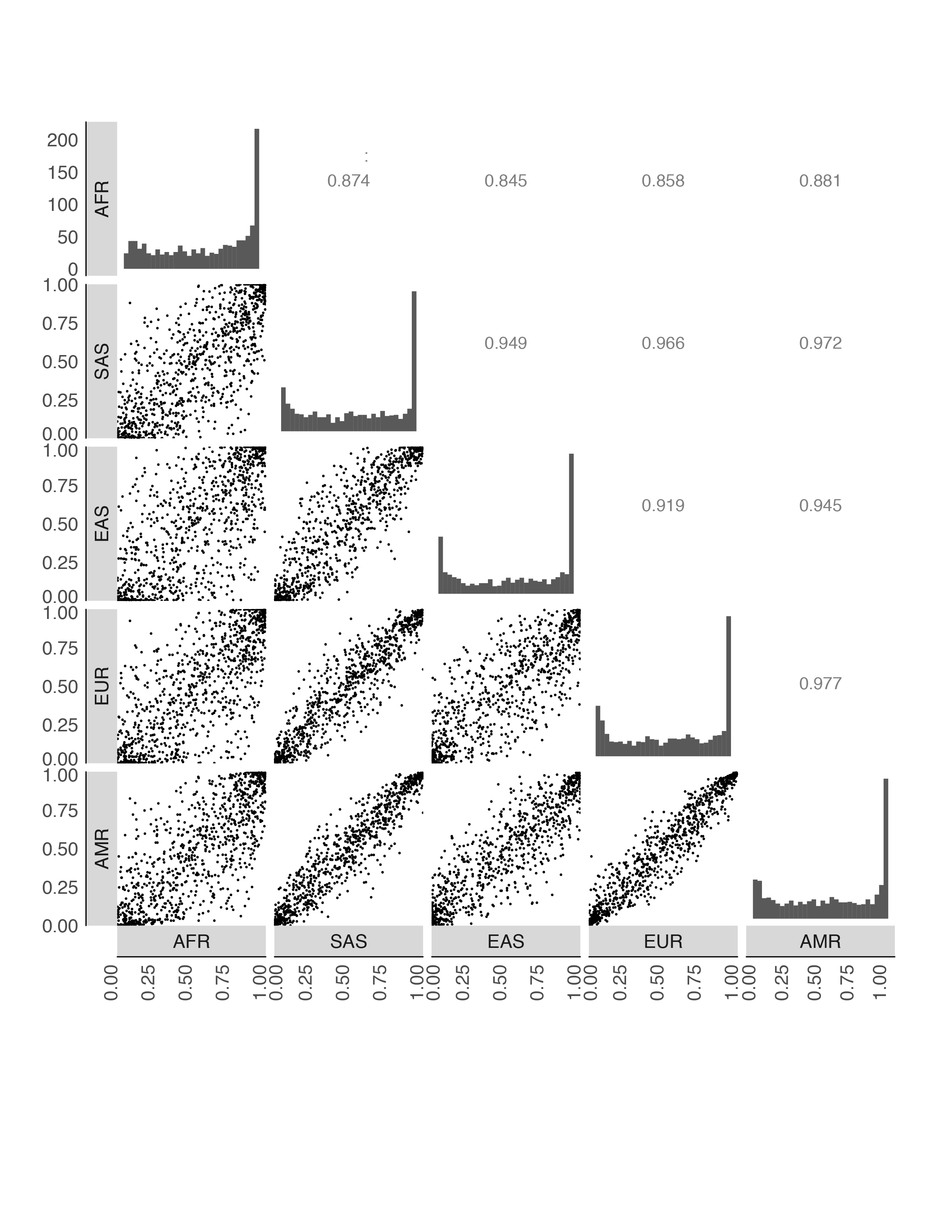
**

Allele frequency correlation plot (lower matrix) among various population groups, and the corresponding Kendall rank correlation coefficient (τ; upper matrix). The diagonal histograms represent the allele frequency spectrum in each population. Abbreviations for each population and continental group are defined in the Methods.

**Figure S7. Phylogenetic tree based on non-reference insertion variants.**

**
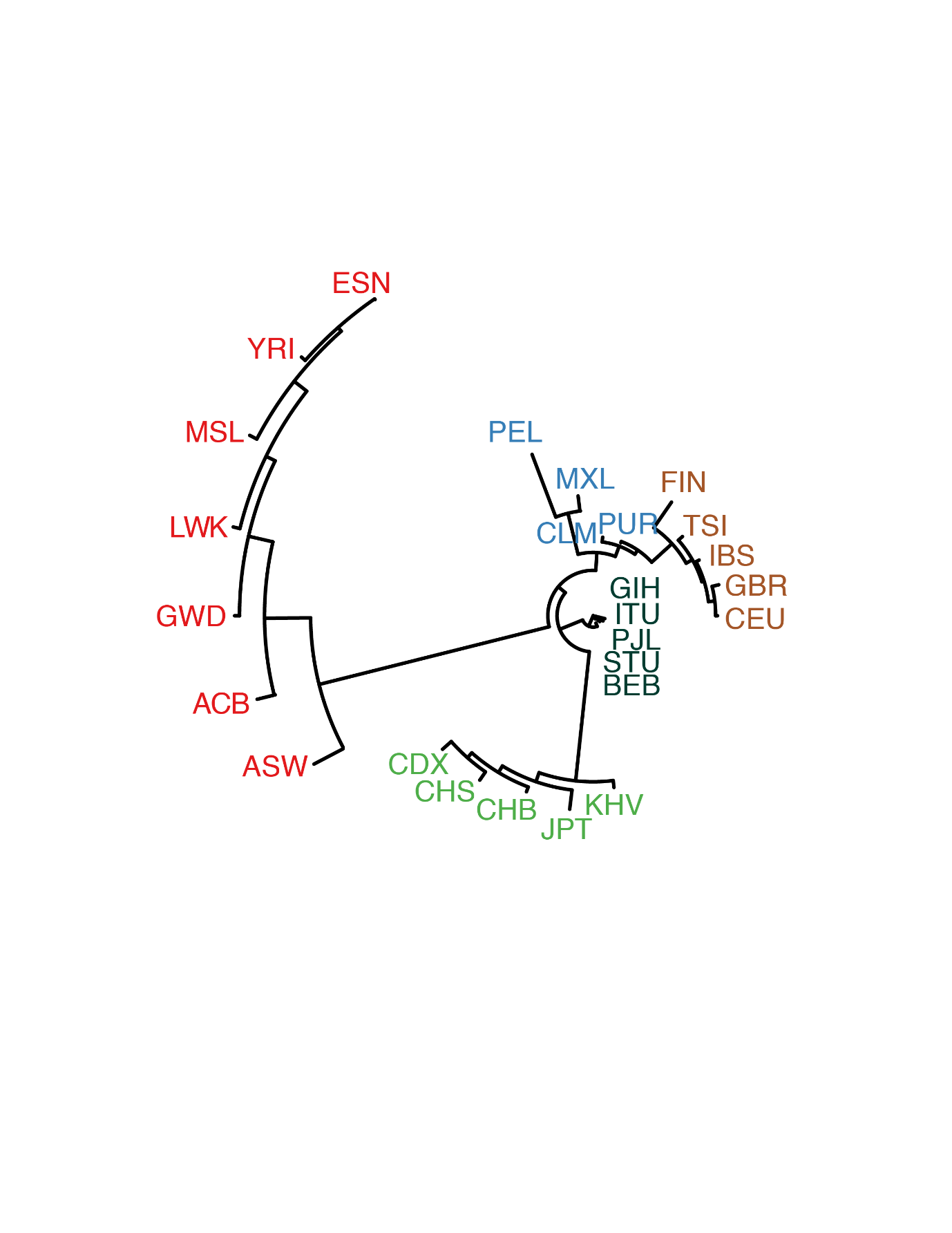
**

The unrooted neighbor joining tree was constructed based on population differentiation (*F*_ST_) calculated using the biallelic genotypes of non-reference insertion variants. Abbreviations for each population and continental group are defined in the Methods.

**Figure S8. Tissues affected by the eQTLs linked to non-reference insertion SVs.**

**
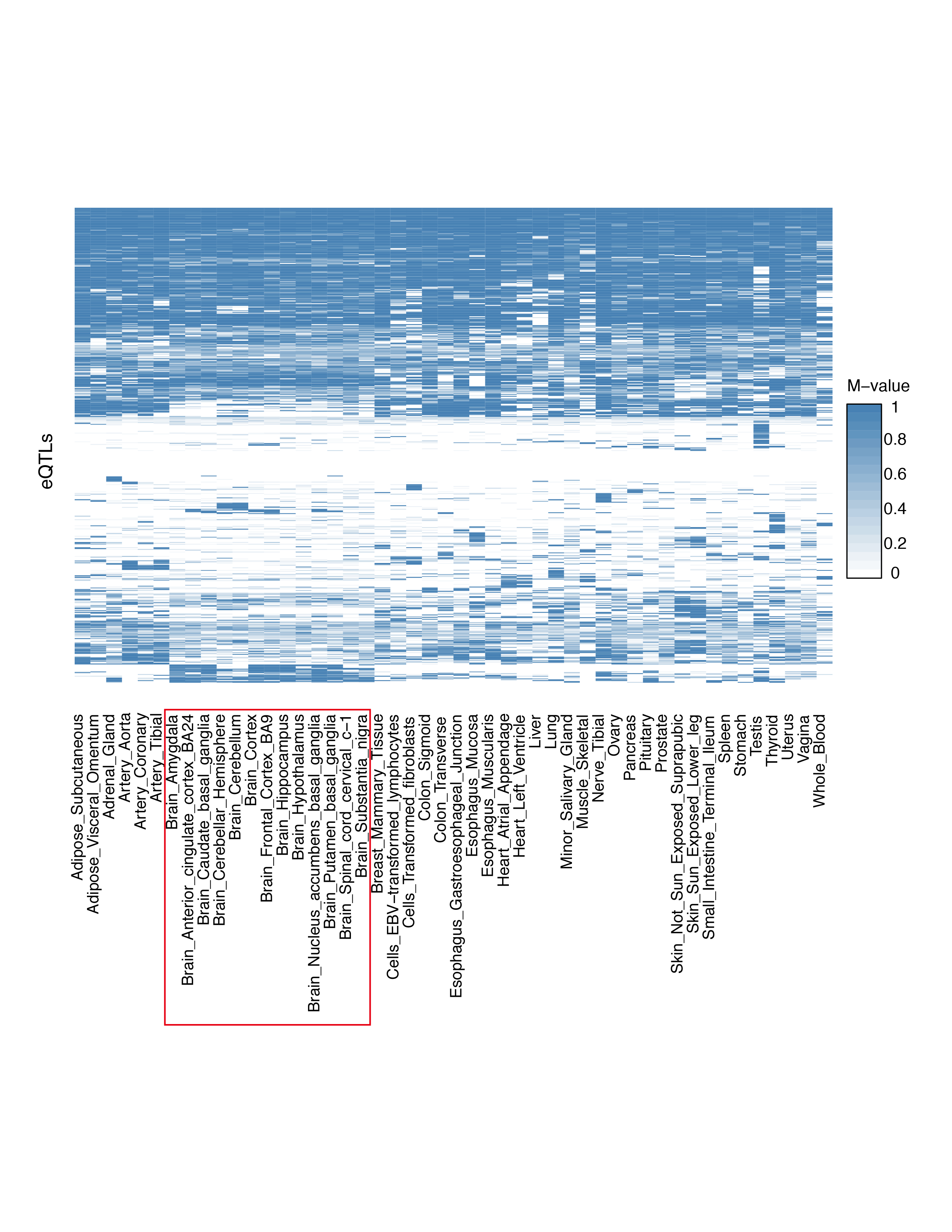
**

Heatmap of the m-values of eQTLs that were in high linkage disequilibrium (*r^2^* > 0.8) and at closest distance to non-reference insertion SVs across 48 tissues. The m-value represents the posterior probability of eQTLs exerting effects in multiple tissues assayed in the GTEx project. The red box indicates 13 brain tissues.

**Figure S9. Haplotype association analysis of I_709 and *GOLIM4*.**

**
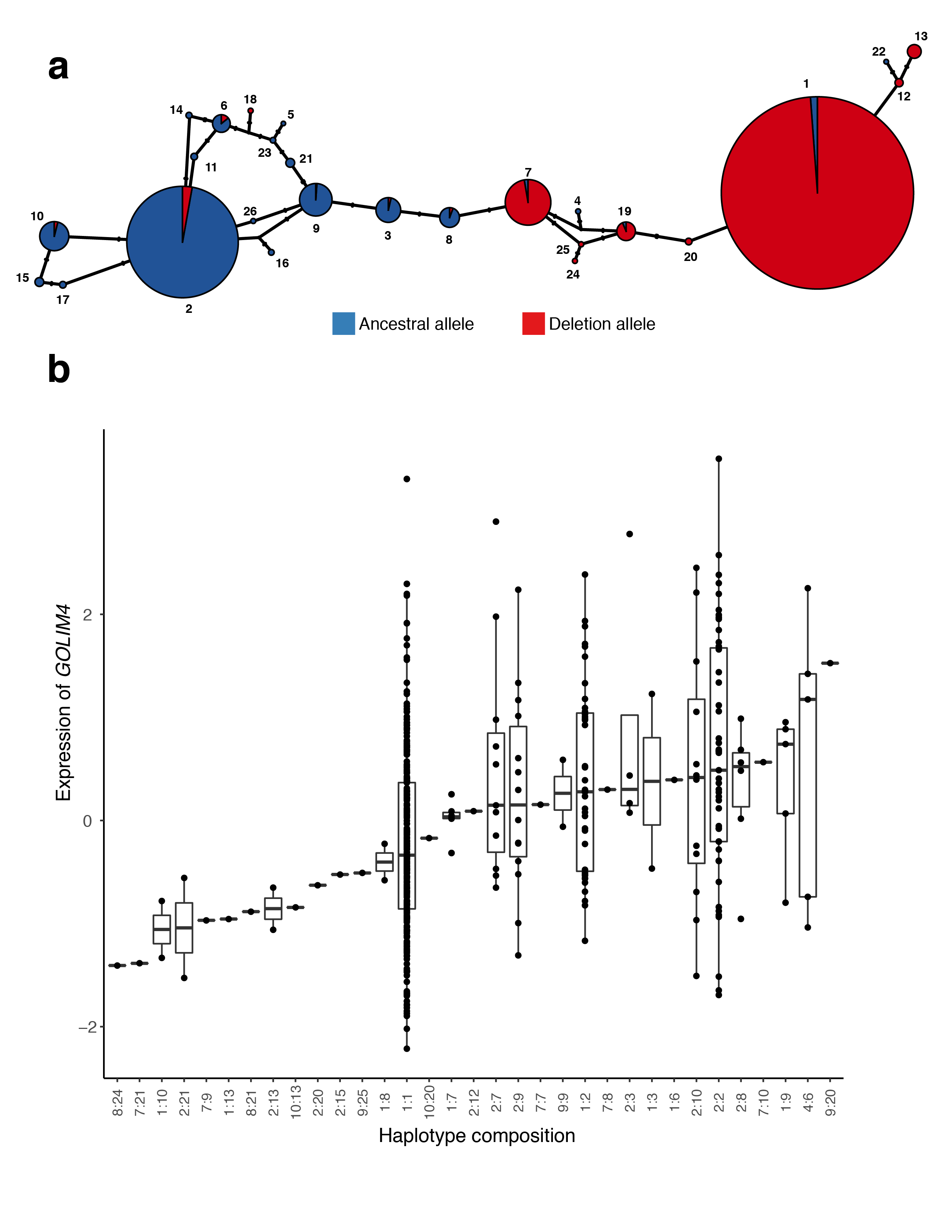
**

(a) Haplotype network of all population groups from the 1000 Genomes Project. The haplotypes are assigned using 18 SNPs within the linkage disequilibrium structure of I_709. Haplotype identifiers are denoted by numbers. (b) Expression patterns *GOLIM4* according to the composition of haplotypes encompassing the I_709 variant. The major haplotype compositions (1:1, 1:2, and 2:2 haplotypes) exhibited significant associations with the expression patterns (β = 0.27, SE = 0.04, p-value = 8.9 × 10^-9^).
